# 2349 The role of interleukin-23 in human melanoma

**DOI:** 10.1017/cts.2018.135

**Published:** 2018-11-21

**Authors:** Aditi Jani, Sandeep Chaudhary, Zorica Janjetovic, Mohammad A. Sherwani, Nabiha Yusuf, Andrzej Slominski, Mohammad Athar, Craig Elmets

**Affiliations:** University of Alabama at Birmingham

## Abstract

OBJECTIVES/SPECIFIC AIMS: Interleukin-23 (IL-23) promotes differentiation of naïve T-cells into Th17 cells, which drive the pathogenesis of autoinflammatory conditions such as psoriasis. IL-23-neutralizing antibody therapies are now in use for treatment of psoriasis, with promising results. Studies in mice have shown that IL-23 plays a role in inhibiting the growth, progression, and metastasis of melanomas. Thus, therapeutic neutralization of IL-23 in patients may inadvertently increase their susceptibility to development of melanoma. In this study, we aim to characterize expression of IL-23 receptors (IL-23R) in human melanocytes and melanoma cells and tissue and to study the effect of IL-23 on growth, proliferation, and tumorigenicity of these cells. METHODS/STUDY POPULATION: IL-23R expression was characterized using immunofluorescence staining, Western blot, and flow cytometric analysis. Response of melanoma and melanocytes to recombinant IL-23 treatment will be studied through similar methods in addition to assays of cell proliferation and tumorigenicity. RESULTS/ANTICIPATED RESULTS: Preliminary immunofluorescence staining and flow cytometry results indicate that both human melanoma and primary melanocytes express IL-23 receptors. Western blot analysis showed that melanoma cell line A375 expressed nearly twice the amount of IL-23R versus normal melanocytes (*p*<0.05). Based on previous studies, we anticipate that addition of recombinant IL-23 to cultures of melanoma will reduce proliferative potential, and we expect similar addition to normal melanocytes will increase DNA repair mechanisms. DISCUSSION/SIGNIFICANCE OF IMPACT: In showing that human melanocytes and melanoma cells express IL-23 receptors, and potentially showing the inhibitory effect of IL-23 in the development of melanocytic neoplasms, our findings imply that using IL-23 neutralizing therapies may increase risk of developing melanoma, especially in patients who are already susceptible. As such, these therapies must be used with great care in these patients.

